# Assessment of cone beam computed tomography for determining position and prognosis of interradicular mini-implants

**DOI:** 10.1590/2177-6709.27.5.e222190.oar

**Published:** 2022-11-28

**Authors:** Eroncy Souto BATISTA, Ademir FRANCO, Mariana Quirino Silveira SOARES, Monikelly do Carmo Chagas NASCIMENTO, José Luiz Cintra JUNQUEIRA, Anne Caroline OENNING

**Affiliations:** 1 Faculdade São Leopoldo Mandic, Instituto de Pesquisas São Leopoldo Mandic (Campinas/SP, Brazil).

**Keywords:** Cone beam computed tomography, Orthodontic anchorage, Bone screws, Oral imaging

## Abstract

**Objective:**

To investigate the influence of dynamic visualization of cone beam computed tomography (CBCT) scans on orthodontist’s assessment of positioning status and prognosis of interradicular mini-implants (MI).

**Methods:**

Three MI positions were virtually simulated in thirty CBCT volumes: (1) MI 1 mm from the lamina dura (LD), (2) MI touching the LD and (3) MI overlapping the LD. Each position was exposed to orthodontists (n = 35) as panoramic reconstruction, sagittal reconstruction and a sequence of axial slices. Each orthodontist evaluated the MI position (relationship with the LD) and scored the prognosis using a four-point scale (the higher the score, the better the prognosis). Kappa, Friedman and Nemenyi statistics were used.

**Results:**

Statistically significant associations were detected between the prognosis scores and the type of image visualized (*p*<0.05). The dynamic visualization of the CBCT volume (axial slices) was associated with higher scores for prognosis and more reliable evaluation of MI positioning. Inconsistent outcomes were more frequently associated with panoramic and sagittal reconstructions.

**Conclusion:**

The dynamic visualization of axial slices allowed orthodontists to perform better assessment of MI position and considerably affected prognosis judgment.

## RESUMO

**Objetivo:**

Analisar se a avaliação dinâmica de volumes de tomografia computadorizada de feixe cônico (TCFC) influencia a percepção de ortodontistas quanto à determinação da posição e do prognóstico de mini-implantes (MIs) inter-radiculares.

**Material e Métodos:**

Foram selecionados 30 volumes de TCFC, nos quais foram simuladas três formas de instalação de MIs: 1) MI a 1 mm de distância da lâmina dura (LD); 2) MI tocando a LD; e 3) MI sobreposto à LD. As imagens foram examinadas por 35 ortodontistas na forma de reconstruções panorâmicas, reconstruções sagitais e uma sequência de reconstruções axiais. Os examinadores avaliaram a relação entre o MI e a LD, e indicaram o prognóstico dos MIs instalados, usando uma escala de 4 pontos (quanto maior o escore, mais favorável o prognóstico). Os dados foram submetidos à análise estatística, por meio dos testes de Kappa, Friedman e Nemenyi.

**Resultados:**

Houve associação significativa entre o prognóstico, os tipos de imagem e as três formas de inserção dos MIs (*p*<0,05). A visualização dinâmica dos volumes de TCFC (reconstruções axiais) foi associada a maiores escores para o prognóstico e a avaliações mais confiáveis da posição dos MIs. Escores mais inconsistentes e discordantes foram mais associados às reconstruções panorâmicas e sagitais.

**Conclusão:**

A análise do volume tomográfico em reconstruções axiais pode melhorar a avaliação do ortodontista quanto à posição dos MIs inter-radiculares, apresentando impacto significativo na determinação do prognóstico do caso.

## INTRODUCTION

In Orthodontics, specific movements that were challenging in the past, such as distalization and intrusion, became more feasible with the advent of mini-implants (MI).^1^ ^-^ ^3^ In addition to proper biomechanics, the MI reduce anchorage loss.^4^ Clinical risks, however, may occur especially when MI damage the periodontal ligament or adjacent roots^5^ ^,^ ^6^ and trigger external root resorption.^7^ In order to minimize root resorption and promote proper bone anchorage, MI should be placed at least 1 mm far from the lamina dura.^8^ From the perspective of treatment prognosis, having MI in contact with root and periodontal ligament is one of the main causes of MI failure.^6^ ^,^ ^9^ ^-^ ^13^

Studies with preoperative radiographs were designed to find out safer anatomic regions for MI placement (i.e. regions with more interradicular space),^14^ ^,^ ^15^ but the available space varies between patients due to the broad spectrum of skeletal discrepancies, axial tooth angles and anatomic variations.^14^ Postoperative imaging, on the other hand, might be straight to the point when it comes to the assessment of dentoalveolar damages.^16^ Protocols that include postoperative radiographs, however, may alter clinical judgment about the final position of the MI - without necessarily, increasing certainty.^17^

Image superimposition hinders a clear visualization of dentomaxillofacial structures in two-dimensional images.^11^ ^,^ ^15^ ^,^ ^18^ Differently, three-dimensional imaging, namely cone beam computed tomography (CBCT), improves assessment of the relationship between MI and adjacent teeth and bone.^4^ ^,^ ^16^ Higher radiation dose and cost restrict the use of CBCT^19^ and encourage pertinent questions, such as: considering cost (biological and financial) vs. benefit (clinical contribution), is CBCT justified for the postoperative assessment of MI?

Thus, this study aimed to verify if the analysis of CBCT volumes could have influence on orthodontists’ judgment of MI positioning and prognosis. The null hypothesis was that there would be no association between the determination of MI positioning and prognosis, and the use of two-dimensional reconstructions or CBCT volumes. 

## MATERIAL AND METHODS

### ETHICAL ASPECTS AND STUDY DESIGN

This cross-sectional observational study was carried out with the approval of the institutional committee of ethics in human research (protocol: 3.651.240).

### SAMPLE

Thirty CBCT volumes, stored in DICOM format, were selected from the image database of a public university. All the images were acquired in an i-CAT^®^ device (Imaging Sciences International Inc., Hatfield, PA, USA). The CBCT scans were acquired with a field of view (FOV) of 23 cm x 17 cm, given that this FOV size is frequently used for orthodontic indications, and consequently, it is also used for mini-implant planning. The inclusion criteria consisted of CBCT volumes of males and females taken with the same acquisition and reconstruction settings, to avoid the inclusion of bias due to differences in noise level and/or spatial resolution. The patients included should be eligible for orthodontic treatment with MI and should have maxillary first molars and second premolars, as well as sound bone structure in the region. Patients under 18 years of age, with metallic restorations or prosthetic materials in the region or any type of anatomic variation or lesion in the adjacent alveolar bone and maxilla were excluded.

**Figure 1:**
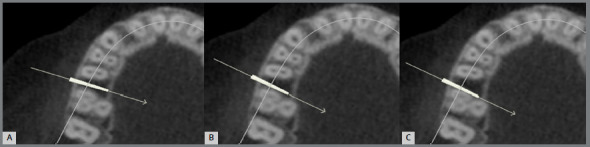
Three scenarios (axial slices) of simulated mini-implant placement: **A**) group “far from the lamina dura”; **B**) group “touching the lamina dura”; **C**) group “overlapping the lamina dura”.

The DICOM files were imported to OnDemand 3D software (Kavo, Biberach an der Riss, Germany). Within the software, MI (1.6 mm x 10 mm) placement was simulated in three groups: MI positioned 1 mm from the lamina dura (LD) (Group “far from the LD”; n = 10); MI “touching the LD” (n = 10); and MI “overlapping the LD” (n = 10) (Fig 1).

**Figure 2:**
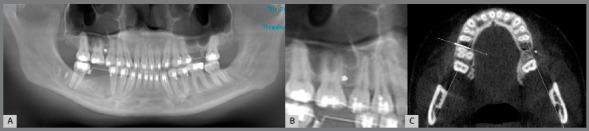
A) Panoramic reconstruction, B) Sagittal reconstruction, C) one of the axial slices that composed the volume. The images in A, B and C were obtained from the simulation “touching the lamina dura”.

The simulation of surgical placement of MI was designed between the maxillary first molar and second premolar, within a distance of 2 mm from the bone crest and perpendicular to the adjacent teeth.^20^ For each simulation, three sets of images were generated: panoramic reconstructions (n = 30) with 20-mm thickness, sagittal reconstructions (aligning sagittal reference line to the arch and increasing the thickness to 20 mm) (n = 30), and videos (n = 30) depicting the navigation through sequential axial slices (Fig 2). The navigation started from the contact point between first molar and second premolar to the apical region of both teeth. The sets of images were coded to ensure a blind process, and the examiners were provided with these sets, together with instructions for visualization and analysis.

### IMAGE ANALYSIS

Thirty-five orthodontists were invited to perform image analysis (mean age of 34 years, SD 5.08, range 27- 43 years). The inclusion criteria for the examiners consisted of previous experience with history of orthodontic practice and knowledge of MI therapeutics for at least three years. The set of 10 images (of each type of simulation) was considered the investigative tool, while the orthodontists (observers) were considered the sample (with quantified repetitions). In this context, the quantity of examiners was set by means of sample size calculation (G*power, Dusseldorf, Germany) considering test power of 0.80 (β=0.20) to detect medium size effects^34^ ^,^ ^35^ of dz = 0.50 at a significance level of 0.05 (α=0.05).

Each orthodontist blindly and randomly analyzed the complete set of images (n=90). Image analysis was carried out in three time intervals: (1) analysis of 30 panoramic reconstructions; (2) analysis of 30 sagittal reconstructions; and (3) analysis of 30 CBCT volumes recorded as axial navigation videos. There was an interval of 10 days between analyses. From one analysis to another, the sequence of patients was randomized, to avoid memorization.

The examiners were instructed to perform the analysis on computer screens of at least 15-in, in a quiet place with reduced ambient light. They classified the position of the MI in relation to the adjacent alveolar LD into four categories: far from the LD (1), touching the LD (2), overlapping the LD (3) and impossible to determine (4). Additionally, the orthodontists were requested to make inferences about the prognosis according to the scale previously used by Oba et al.^17^: definitely not favorable (1), probably not favorable (2), probably favorable (3) and definitely favorable (4). Ten days after the final analysis, the examiners reanalyzed 30% of the sample (nine images from each group) to assess intra-examiner reproducibility.

### DATA ANALYSIS

Study outcomes were assessed based on the descriptive data and frequency of answers for the classification of MI prognosis and positioning. The chi-square test was used to verify the association between the mean judgment scores and the type of image. The agreement between examiners’ classification (MI relationship with the LD) and simulated position was assessed by means of Kappa statistics (95% confidence interval). Considering the answer “impossible to determine”, sagittal and panoramic reconstructions were compared with the CBCT volume, using McNemar test. For the analysis of scores based on prognosis, the median score of each image type and each examiner was considered. Comparisons between types of images and MI positioning were accomplished with Friedman’s and Nemenyi’s tests for multiple comparisons. Kappa statistics assessed intra-examiner reproducibility. Data analysis was performed with R software (R foundation, Vienna, Austria) with significance level of 5%.

## RESULTS

### ASSESSMENT OF MI POSITIONING

Table 1 presents the outcomes of examiner scores for MI positioning. The agreement between examiner scores and the real MI position was fair for the three image types^36^, with Kappa equal to 0.26, 0.27 and 0.39, for panoramic and sagittal reconstructions, and CBCT volumes, respectively. The frequency of “impossible to determine” answers was significantly higher in panoramic and sagittal reconstructions (10.4%), compared with the CBCT volume (3.2%) (*p* < 0.05).

**Table 1:** Agreement between examiner’s scores for mini-implant positioning and the real position established in each group.

Image type	Mini-implant positioning	Examiner’s scores				Total
		Far from the LD	Touching the LD	Overlapping the LD	Impossible to determine	
		n (%)	n (%)	n (%)	n (%)	n (%)
PANORAMIC RECONSTRUCTIONS	Far from LD	273 (78.0%)	37 (10.6%)	20 (5.7%)	20 (5.7%)*^1^	350 (100.0%)
	Touching the LD	141 (40.3%)	97 (27.7%)	64 (18.3%)	48 (13.7%)*^2^	350 (100.0%)
	Overlapping the LD	120 (34.3%)	110 (31.4%)	79 (22.6%)	41 (11.7%)*^3^	350 (100.0%)
Total		534 (50.9%)	244 (23.2%)	163 (15.5%)	109 (10.4%)	1050 (100.0%)
Weighted Kappa (95% CI)		0.26 (0.22-0.30)				
SAGITTAL RECONSTRUCTIONS	Far from LD	255 (72.9%)	48 (13.7%)	24 (6.9%)	23 (6.6%)*^4^	350 (100.0%)
	Touching the LD	154 (44.0%)	82 (23.4%)	74 (21.1%)	40 (11.4%)*^5^	350 (100.0%)
	Overlapping the LD	93 (26.6%)	113 (32.3%)	98 (28.0%)	46 (13.1%)*^6^	350 (100.0%)
Total		502 (47.8%)	243 (23.1%)	196 (18.7%)	109 (10.4%)	1050 (100.0%)
Weighted Kappa (95% CI)		0.27 (0.22-0.32)				
CBCT VOLUME	Far from LD	304 (86.9%)	27 (7.7%)	14 (4.0%)	5 (1.4%)	350 (100.0%)
	Touching the LD	191 (54.6%)	71 (20.3%)	67 (19.1%)	21 (6.0%)	350 (100.0%)
	Overlapping the LD	55 (15.7%)	146 (41.7%)	141 (40.3%)	8 (2.3%)	350 (100.0%)
Total		550 (52.4%)	244 (23.2%)	222 (21.1%)	34 (3.2%)	1050 (100.0%)
Weighted Kappa (95% CI)		0.39 (0.35-0.43)				

*Different from the percentage of “impossible to determine” answers for image analysis through CBCT volume for the same mini-implant positioning (*p*≤0.05). Comparisons for the same position within CBCT volume: ^1^p<0.0001; ^2^p=<0.0001; ^3^p<0.0001; ^4^p<0.0001; ^5^p<0.0001; ^6^p<0.000. LD: lamina dura.

Analyzing the scores (1 - far from the LD, 2 - touching the LD, 3 - overlapping the LD, 4 - impossible to determine) from another perspective, when “impossible to determine” (score 4) was considered during data analysis, lack of difference was found between image types (Table 2). Thus, the score data were analyzed by removing the score 4. In that sense, the higher the score, the higher would be the proximity to the LD. As a result, statistically significant differences were detected between the image types for MI “overlapping the LD” (Table 3) (*p*<0.05). In particular, higher scores (high proximity) were detected within the CBCT volumes, compared with the panoramic and sagittal reconstructions (*p*<0.05).

**Table 2:** Mode and median (minimum and maximum) scores (1-4) of mini-implant positioning distributed per image type.

Image type	Mini-implant positioning			p
	Far from LD	Touching the LD	Overlapping the LD	
Panoramic reconstruction	1; 1.0 (1.0; 3.0) ^Aa^	1; 2.0 (1.0; 4.0) ^Ba^	1; 2.0 (1.0; 4.0) ^Ba^	< 0.0001
Sagittal reconstruction	1; 1.0 (1.0; 2.5) ^Aa^	1; 2.0 (1.0; 4.0) ^Ba^	2; 2.0 (1.0; 4.0) ^Ba^	< 0.0001
CBCT volume	1; 1.0 (1.0; 2.5) ^Aa^	1; 1.5 (1.0; 3.0) ^Ba^	2; 2.0 (2.0; 3.0) ^Ca^	< 0.0001
*p*	0.8187	0.1242	0.1160	

Scores: 1 - far from the LD , 2 - touching the LD , 3 - overlapping the LD, 4 - impossible to determine. Different letters indicate statistically significant differences between outcomes (uppercase for horizontal comparisons and lowercase for vertical comparisons) (*p*≤0.05).

**Table 3:** Mode and median (minimum and maximum) scores (1-3) of mini-implant positioning distributed per image type, not considering the “impossible to determine” (4) answer.

Image type	Mini-implant positioning			p
	Far from the LD	Touching the LD	Overlapping the LD	
Panoramic reconstruction	1; 1.0 (1.0; 1.5) ^Aa^	1; 2.0 (1.0; 3.0) ^Ba^	1; 2.0 (1.0; 3.0) ^Ba^	< 0.0001
Sagittal reconstruction	1; 1.0 (1.0; 1.5) ^Aa^	1; 1.5 (1.0; 3.0) ^Ba^	2; 2.0 (1.0; 3.0) ^Cab^	< 0.0001
CBCT volume	1; 1.0 (1.0; 2.5) ^Aa^	1; 1.0 (1.0; 3.0) ^Aa^	2; 2.0 (2.0; 3.0) ^Bb^	< 0.0001
*p*	0.8607	0.1840	0.0019	

Scores: 1 - far from the LD , 2 - touching the LD , 3 - overlapping the LD. Different letters indicate statistically significant differences between outcomes (uppercase for horizontal comparisons and lowercase for vertical comparisons) (*p*≤0.05).

### ASSESSMENT OF MI PROGNOSIS

Table 4 shows a significant association between the position of the MI and examiners’ scores for prognosis, considering the three image types (*p*<0.05). An in-depth look at MI that were far from the LD revealed a higher percentage of definitely favorable answers obtained from CBCT volumes (61.7%) compared with panoramic (57.1%) and sagittal (47.7%) reconstructions. CBCT volumes also revealed a higher percentage of definitely unfavorable prognoses when the MI overlapped the LD (21.7%). 

**Table 4:** Agreement between examiner’s scores for mini-implant prognosis and their position in the three image types.

Image type	Mini-implant position	Prognosis				Total
		Definitely not favorable	Probably not favorable	Probably favorable	Definitely favorable	
		n (%)	n (%)	n (%)	n (%)	n (%)
PANORAMIC RECONSTRUCTIONS	^1.^Far from LD*	5 (1.4%)	36 (10.3%)	109 (31.1%)	200 (57.1%)	350 (100.0%)
	^2.^Touching the LD*	14 (4.0%)	106 (30.3%)	159 (45.4%)	71 (20.3%)	350 (100.0%)
	^3.^Overlapping the LD*	37 (10.6%)	119 (34.0%)	128 (36.6%)	66 (18.9%)	350 (100.0%)
Total		56 (5.3%)	261 (24.9%)	396 (37.7%)	337 (32.1%)	1050 (100.0%)
*p*		< 0.0001				
SAGITTAL RECONSTRUCTIONS	^4.^Far from LD*	4 (1.1%)	51 (14.6%)	128 (36.6%)	167 (47.7%)	350 (100.0%)
	^5.^Touching the LD*	36 (10.3%)	82 (23.4%)	146 (41.7%)	86 (24.6%)	350 (100.0%)
	^6.^Overlapping the LD*	38 (10.9%)	125 (35.7%)	146 (41.7%)	41 (11.7%)	350 (100.0%)
Total		78 (7.4%)	258 (24.6%)	420 (40.0%)	294 (28.0%)	1050 (100.0%)
*p*		< 0.0001				
CBCT VOLUMES	Far from LD	6 (1.7%)	16 (4.6%)	112 (32.0%)	216 (61.7%)	350 (100.0%)
	Touching the LD	12 (3.4%)	73 (20.9%)	182 (52.0%)	83 (23.7%)	350 (100.0%)
	Overlapping the LD	76 (21.7%)	151 (43.1%)	99 (28.3%)	24 (6.9%)	350 (100.0%)
Total		94 (9.0%)	240 (22.9%)	393 (37.4%)	323 (30.8%)	1050 (100.0%)
*p*		< 0.0001				

*Differ from the analyses performed for the CBCT volume for the same MI positioning (*p*≤0.05). Comparisons for the same MI positioning for the CBCT volume: ^1^ *p*=0.0378; ^2^ *p*=0.0332; ^3^ *p*<0.0001; ^4^ *p*<0.0001; ^5^ *p*=0.0009; ^6^ *p*<0.0001.

Comparisons based on prognosis scores are found in Table 5 (the higher the score, the better the prognosis). Compared with reconstructions, stronger and statistically significant associations were found when the MI was positioned far from the LD / overlapping the LD and observed through CBCT volumes (p<0.05). In other words, only in CBCT volumes there was a statistical distinction of the prognosis among the three MI positions.

**Table 5:** Mode and median (minimum and maximum) comparisons between the prognoses scored by the examiners, according to the position of the mini-implant and the image type.

Image type	Mini-implant positioning			p
	Far from LD	Touching the LD	Overlapping the LD	
Panoramic reconstruction	4; 4.0 (2.5; 4.0) ^Bab^	3; 3.0 (2.0; 4.0) ^Aa^	3; 2.5 (2.0; 4.0) ^Ab^	< 0.0001
Sagittal reconstruction	4; 3.0 (2.5; 4.0) ^Ba^	3; 3.0 (2.0; 4.0) ^Aa^	3; 3.0 (1.0; 3.5) ^Ab^	< 0.0001
CBCT volume	4; 4.0 (3.0; 4.0) ^Cb^	3; 3.0 (2.0; 4.0) ^Ba^	2; 2.0 (1.0; 3.0) ^Aa^	< 0.0001
*p*	0.0134	0.5220	0.0009	

Different letters indicate statistically significant differences between outcomes (uppercase for horizontal comparisons and lowercase for vertical comparisons) (*p*≤0.05).

### INTRAEXAMINER REPRODUCIBILITY

For MI positioning, Kappa agreement reached 0.523 (moderate agreement), 0.546 (moderate agreement) and 0.709 (substantial agreement) for the panoramic reconstruction, sagittal reconstruction and CBCT volume, respectively. For the prognosis, all the reproducibility tests resulted moderate, Kappa reached 0.543, 0.546 and 0.546 for the same image types above mentioned, respectively.

## DISCUSSION

In the scientific literature, there is a scarcity of studies designed to find out the influence of CBCT on the treatment plan and prognosis in Orthodontics.^19^ According to the American Academy of Orthodontics (AAO) and the American Academy of Oral and Maxillofacial Radiology (AAOMR), CBCT is indicated for the assessment of potential sites for MI placement.^21^ The Sedentex-CT guidelines, on the other hand, established European standards suggesting the MI does not necessarily require CBCT visualization, except in critical anatomic sites.^22^ Scientific studies have demonstrated that two-dimensional imaging might not be sufficient for clear visualization of MI surgical sites - and have recommended CBCT instead.^16^ The present study corroborated these findings by suggesting that safe diagnosis of MI positioning and speculated prognosis may be obtained from the dynamic visualization of the CBCT volume.

CBCT had a significant impact on orthodontists’ judgment of MI position, but mostly on prognosis. Notably, the role of CBCT was especially relevant in more challenging images (i.e. MI touching or overlapping the LD). Because close contact between MI and dental roots is a risk factor for failure,^1^ ^,^ ^11^ ^,^ ^23^ CBCT could figure as a proper tool to predict MI failure (not favorable prognosis) in clinical practice, by accurately revealing MI in close contact with the LD. It is worth emphasizing that when the periodontal ligament is damaged (MI overlapping the LD), root resorption is an inevitable outcome if the MI is not immediately removed.^24^

Apparently, CBCT analyses might not be necessary for installing MI with anchorage in the palate (close to the median maxillary suture).^25^ Lateral radiographs may enable correct and reliable assessment of bone thickness prior to surgery, while CBCT would be of major value in complex cases with borderline anatomic features.^26^ ^,^ ^27^ This is why the sample collected in the present study consisted of MI anchored in the alveolar process.

For the outcomes related to MI positioning, CBCT volumes did not necessarily increase the agreement between real (known) and judged (examiners’) positions. The number of “impossible to determine” answers, however, was considerably lower in CBCT volumes - suggesting that the examiners were more confident while navigating through the image slices. Moreover, intra-examiner agreement in CBCT analysis was higher (0.709) compared with the analysis of two-dimensional reconstructions (panoramic: 0.523 and sagittal: 0.546). The low agreement rates after reproducibility tests might be explained by the fact that orthodontists are not ideally familiar with advanced imaging techniques as other dental specialists, such as oral radiologists. With different samples of examiners, different outcomes could be obtained. Further investigations should be conducted on this point.

Despite the benefits of CBCT, orthodontists must be aware of the potential risk of biological effects associated to the higher radiation dose of this imaging modality, in comparison with conventional two-dimensional radiographies.^19^ Patient-customized protocols optimized for their therapeutic needs are corroborated by the recent ALADAIP (As Low as Diagnostically Acceptable being Indication-oriented and Patient-specific) principle.^28^ ^,^ ^29^ In practice, ALADAIP may help to guide orthodontists toward the best decisions for each patient - both relative to the use of CBCT for diagnosis and MI treatment planning or for prognostic follow-up.

The panoramic and sagittal reconstructions, which counterbalanced the dynamic visualization of the CBCT volume in the present study, were used to simulate, to some extent, radiographs frequently used for evaluation of MI, i.e., panoramic and periapical radiographs. This could represent a limitation of the present study. From a technical perspective these panoramic and sagittal (“periapical”) reconstructions were not essentially real two-dimensional radiographs, mainly due to geometric projection features (e.g. incidence angle, superimposition of structures). They were obtained from the superimposition of several slices in a single plane of 20-mm thickness^30^, and slightly resembled the quality and general aspect of anatomic structures of true radiographs. Moreover, from a radiation protection perspective, image acquisition in real patients using three modalities is not justified. Hence, the simulated scenario emerged as a feasible alternative to enable this study. Another limitation of the study was the simulated MI placement, which, on the other hand, was performed to ensure control and standardization of the final images presented to the examiners. Prospectively simulating real MI in patients would be impossible for ethical reasons, particularly in situations of clinical failure. Another limitation of the present study should be mentioned, regarding the large FOV and low spatial resolution (voxel size 0.3mm), both of which could, to some extent, jeopardize detailed analysis.^28^ ^,^ ^31^ ^,^ ^32^ From a clinical perspective, the CBCT scans requested for orthodontic proposals (e.g. cephalometric analysis, evaluation of airways or orthodontic-surgical cases) are not the types optimally used for MI analysis. These conditions (large FOV and voxel size), however, have been used in research involving simulated MI.^33^

Future studies in the field could be designed to develop protocols with smaller FOVs and restricted radiation doses as alternatives toward optimization of the diagnosis and estimated prognosis of patients treated with MI anchored in the maxillary premolar/molar region. Justification and optimization continue to be the keywords for the use of CBCT for MI placement. Orthodontists must be prepared to balance their clinical decisions supported with evidence-based findings. 

## CONCLUSION

» Compared with panoramic and sagittal reconstructions, the dynamic visualization of CBCT volumes improved the judgment of orthodontists about the surgical site for MI placement.
» Analyses of MI position performed on static reconstructions instead of volume navigation led to more uncertainty.
» CBCT visualization of MI positioning significantly impacted the prognosis judgment by the orthodontists.
